# Supplementary material for: The genome sequence of a leafhopper, Allygidius commutatus (Fieber, 1872) (Hemiptera: Cicadellidae)
Source: Wellcome Open Res. 2025 Sep 18;10:521. [Version 1] doi: 10.12688/wellcomeopenres.24880.1 (PMC12824487; doi:10.12688/wellcomeopenres.24880.1)
Supplement: Peer review history

# Reviewer report: Reviewer response for version 1

**DOI:** 10.21956/wellcomeopenres.27405.r140091

**Published:** 2026-02-27

**Authors:** Annabel Whibley

**Affiliations:** 1 Bragato Research Institute, Lincoln, New Zealand; 2 The University of Auckland, Auckland, New Zealand

 Crowley and colleagues report the haplotype-resolved genome assembly of a female leafhopper, *Allygidius commutatus* using the Darwin Tree of Life pipelines and reporting templates. Specimen ID was confirmed by barcoding.  Over 98.9% of the haplotype 1 could be assigned to one of 10 chromosomal scaffolds.  No annotation has been released. The assembly quality is excellent, with comprehensive reporting of methods, metadata, public release of resources and extensive evaluation of the assembly.

Are sufficient details of methods and materials provided to allow replication by others?

Yes

Is the rationale for creating the dataset(s) clearly described?

Yes

Are the datasets clearly presented in a useable and accessible format?

Yes

Are the protocols appropriate and is the work technically sound?

Yes

Reviewer Expertise:

Bioinformatics Genomics

I confirm that I have read this submission and believe that I have an appropriate level of expertise to confirm that it is of an acceptable scientific standard.

# Reviewer report: Reviewer response for version 1

**DOI:** 10.21956/wellcomeopenres.27405.r143071

**Published:** 2026-01-21

**Authors:** Arun Arumugaperumal

**Affiliations:** 1 Department of Biotechnology, Rajalakshmi Engineering College, Thandalam, Chennai, 602105, India

The authors have sequenced the genome of *Allygidius commutatus*. The genome assembly reported here is of size 1796.07 Mb, spread among 10 chromosome molecules. Since a female specimen has been used for sequencing, the X chromosome has been identified. The mitochondrial genome of length 17.75 kb has also been assembled. The authors have followed the Darwin tree of life project standard protocols for sequencing the genome of the insect. The assembly has a high N50 value and BUSCO completeness of 97.4%. The genome annotation has not been released yet through EMBL. The data note can be indexed.

Are sufficient details of methods and materials provided to allow replication by others?

Yes

Is the rationale for creating the dataset(s) clearly described?

Yes

Are the datasets clearly presented in a useable and accessible format?

Yes

Are the protocols appropriate and is the work technically sound?

Yes

Reviewer Expertise:

Bioinformatics; Genomics

I confirm that I have read this submission and believe that I have an appropriate level of expertise to confirm that it is of an acceptable scientific standard.

# Reviewer report: Reviewer response for version 1

**DOI:** 10.21956/wellcomeopenres.27405.r135012

**Published:** 2025-10-30

**Authors:** Valeria Trivellone

**Affiliations:** 1 University of Illinois, Urbana-Champaign, Illinois, USA

I have a few minor suggestions to improve the *Species Taxonomy* and *Background* sections. See below.

 Species Taxonomy : The author’s name should be placed in parentheses, as Fieber originally described the species in a different genus.

 Background : 

 1- Please provide a size range for the species to substantiate the statement:" *Allygidius commutatus* is a relatively small leafhopper."

 2- To support the statement: “one of 13 genomes available for the family Cicadellidae as of August 2025” it would be helpful to include additional information at the subfamily and tribe levels. Indicating only the number of genomes available for the entire family Cicadellidae is not sufficiently informative, given the high taxonomic and ecological diversity within this group.

Are sufficient details of methods and materials provided to allow replication by others?

Yes

Is the rationale for creating the dataset(s) clearly described?

Yes

Are the datasets clearly presented in a useable and accessible format?

Yes

Are the protocols appropriate and is the work technically sound?

Yes

Reviewer Expertise:

ecology and evolution of auchenorrhyncha and phytoplasma associations

I confirm that I have read this submission and believe that I have an appropriate level of expertise to confirm that it is of an acceptable scientific standard.
